# Lipidomics of orchid seeds: from development to storage

**DOI:** 10.1111/plb.70295

**Published:** 2026-09-23

**Authors:** C. C. Custódio, N. B. Machado‐Neto, L. Colville, P. T. Seaton, H. W. Pritchard, T. R. Marks

**Affiliations:** ^1^ Agronomy College Unoeste Campus II Presidente Prudente Brazil; ^2^ Royal Botanic Gardens, Kew Ardingly UK; ^3^ Chinese Academy of Sciences Kunming Institute of Botany Kunming P. R. China

**Keywords:** Fatty acids, fruit growth, germination, orchidaceae, seed maturation, storage

## Abstract

- Orchid seeds primarily contain lipid reserves, yet details of their deposition, in quantity and quality, remain unknown. Deposition begins as carbohydrates, which are eventually converted into fatty acids during seed development. This research aimed to dissect the pattern of lipid deposition in seeds of *Dactylorhiza fuchsii* and *Disa uniflora*, from pollination to seed storage, including at five stages of seed maturation, germination capacity and relationships among fatty acids (FAs).
- The capsules were harvested, cleaned and measured. Extracted seeds were analysed using differential scanning calorimetry (DSC), moisture content determinations, *in vitro* germination tests in three different media and seed viability. A portion of the seeds was dried to analyse lipid content and types via Gas Chromatography–Mass Spectrometry (GC–MS).
- While *Dact. fuchsii* seeds showed a high percentage of dry matter at the end of maturation, *Disa uniflora* had a lower one. Lipid accumulation profiles differed. The proportions of saturated and unsaturated FA varied, with *Disa uniflora* having more saturated FA than *Dact. fuchsii*. Seeds began to germinate before the midpoint of capsule maturation, and germination increased with maturation time in both species, with *Disa uniflora* seeds reaching full germination at capsule dehiscence. Conversely, *Dact. fuchsii* seeds did not germinate because the seed carapace may hinder germination. Nevertheless, at capsule maturity, seeds from both species were highly viable based on the tetrazolium test.
- Mature seeds of both species were desiccation‐tolerant (4.5% RH) and could be stored dry at 5 °C for 3 months without loss of viability, suggesting the possibility of long‐term seed banking for conservation.

## Graphical abstract

The study tracked lipid changes from pollination to storage in two orchid species. Although both were desiccation‐tolerant and storable, differences in saturated and unsaturated fatty acid proportions during development led to different moisture contents and germination capacities.

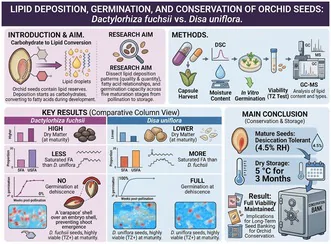

## INTRODUCTION

Orchids are valued as both ornamentals and medicinal plants and are intensively collected, leading to the transfer of wild germplasm into private collections (Yam *et al*. 2009). Orchid species populations are increasingly driven to extinction by habitat degradation and the increasing use of land for crops, grazing and mining (Fay 2018). In the floral world, orchids are considered one of the largest families, with 703 genera and over 30,000 species distributed across five subfamilies: Apostasioideae, Cypripedioideae, Epidendroideae, Orchidoideae and Vanilloideae (Chase *et al*. 2015; Hassler 2026). Orchids are important ornamental flowers (Hinsley *et al*. 2015), and some genera have many hybrids and breeding species, supporting a valuable market for cut fresh flowers; others have been used for medicinal/food purposes, with dried pseudobulbs/tubers traded globally, worth over 1 billion dollars (Yuan *et al*. 2021). Orchid seeds produced through controlled pollination serve as the starting material for breeding programmes, yet relatively little is known about their physiology and biochemistry. At maturity, individual orchid seeds are very small (Arditti & Ghani 2000; Fileti *et al*. 2021), globular to prolated spheroid, undifferentiated embryos with relatively few cells and contain no storage organs such as endosperm or cotyledons, with a single, or sometimes two or three, layers of cells as a seed coat (Ghimire *et al*. 2020; Yeung 2022; Lee & Yeung 2023).

Conservation of orchids in seed banks is of great interest (Hay *et al*. 2010; Seaton *et al*. 2010, 2018; Popova *et al*. 2015; Fileti *et al*. 2021; Hengling *et al*. 2021; Francisqueti *et al*. 2024; Singer *et al*. 2024), and potentially very efficient as their very small size means that a large volume of seeds can be stored in just a few containers. Seed availability for conservation can be exceptional, as capsules contain from a few hundred to millions of seeds (Yam & Arditti 2009).

Seeds are among the most interesting biological structures, carrying a complete new entity with reserves to support the seedling as it colonises the environment. However, germination remains poorly understood in many species (Bewley *et al*. 2013). With more than 300,000 flowering plant species (Lughadha *et al*. 2016), only a few agricultural, forestry and model species have been studied thoroughly enough for germination events to be well understood. For orchids (*e.g. Cattleya, Paphiopedilum, Phalaenopsis*), their oily seeds (Fileti *et al*. 2021; Hengling *et al*. 2021; Diantina *et al*. 2022; Francisqueti *et al*. 2024) may have relatively inefficient enzymatic systems for converting storage lipid into soluble sugars (Manning & van Staden 1987; Lee *et al*. 2006, 2008), an essential step in the conversion of germinating seeds into seedlings. Therefore, the quantity and quality of lipid composition, and how it varies during seed and capsule development, has potentially a profound impact on the efficiency of germination. Analysis of *Cymbidium sinense* (Andrews) Willd seeds revealed that they initially contain starch and accumulate lipid as progress is made towards maturation (Yeung *et al*. 1996). The presence of starch appears to be a residue of the seed (embryo in orchids) filling phase, rather than as the final reserve in the seed at maturation, as it is consumed as the embryo matures (Manning & van Staden 1987). Furthermore, orchid plants not infected by mycorrhizae cannot grow on polysaccharides, and attempts to germinate seeds asymbiotically in the absence of simple sugars are not always successful, even with the addition of vitamins, hormones or other substances (Arditti & Ernst 1984). Therefore, fungal colonisation is needed to mobilise reserves at any stage, from seed to early protocorm and then allow seedling growth (Batty *et al*. 2001; Bertolini *et al*. 2011; Rasmussen 2011).

During orchid seed development, three critical steps are essential: (1) histodifferentiation, (2) reserve deposition and (3) drying during maturation (Bewley *et al*. 2013), covering the general processes of morphogenesis and maturation (Baud & Lepiniec 2010). During morphogenesis, the embryo develops through a series of cell divisions, which are controlled to progressively acquire the basic plan of the plant. Within the maturation phase, storage compounds accumulate in the seed (embryo) tissues, and the seed becomes germinable; thereafter, the seed acquires desiccation tolerance, and germinability may be hindered by the development of embryo dormancy, rendering it quiescent (Bewley *et al*. 2013), particularly in temperate terrestrial orchids.

Seed storage reserves typically consist of proteins, complex carbohydrates (primarily starch) and lipids (triglycerides). Carbons in FAs are highly reduced so that the oxidation of oil storage during seed germination releases more than twice the energy of the oxidation of carbohydrates and protein storage on a gram‐per‐gram basis (Graham 2008; Baud & Lepiniec 2010; Bewley *et al*. 2013). The FA biosynthesis process begins at the onset of ripening, coinciding with the completion of embryonic morphogenesis (Baud & Lepiniec 2010). In the case of the orchid *Phalaenopsis amabilis*, the deposition of storage reserves in relation to the seed harvest point, the acquisition of desiccation tolerance, germination and vigour appear to be broadly similar to well‐studied agricultural species (Schwallier *et al*. 2011).

Fatty acid (FA) composition, particularly the unsaturated (UFA) *versus* saturated (SFA) balance, may affect storage behaviour (Walters 2004, 2015; Walters *et al*. 2004). Generally, orchid seeds contain predominantly linoleic acid, a polyunsaturated acid. However, species specificity occurs in the lipid profile in mature seeds; for example, *Dacty. fuchsii* (Druce) Soó contains only two SFAs (palmitic and stearic) whilst *Grammatophyllum speciosum* Blume seed has four SFAs (pentadecanoic, palmitic, stearic, arachidic; Colville *et al*. 2016). In *Cattleya* species, this FA variation is more pronounced, with 4–6 distinct SFAs (Fileti *et al*. 2021; Hengling *et al*. 2021; Francisqueti *et al*. 2024), palmitic acid as the primary SFA and linoleic acid as the UFA. Seeds of *Grammatophyllum speciosum, Dendrobium strebloceras* Rchb.f., *Den. cunninghamii* Lindl, *Gastrodia cunninghamii* Hook.f., *Pterostylis banksii* R.Br. ex A.Cunn. and *Thelymitra nervosa* Colenso exhibited a similar variation in FA (Diantina *et al*. 2022). Whilst ageing can trigger decreases in orchid seed saturated, polyunsaturated, monounsaturated and total fatty acid content, composition alone appears not to be an adequate marker for seed longevity in orchids (Diantina *et al*. 2022; Francisqueti *et al*. 2024). Moreover, how FAs vary in composition during orchid seed development and what the implications are for germination efficiency remains poorly understood.

In this work, two terrestrial species were used as model systems: (a) *Dacty. fuchsii*, a temperate European orchid with a maturation span of 7–8 weeks between pollination and seed release; and (b) *Disa uniflora* P. J. Bergius, a subtropical terrestrial (South African) species, with a 4–6‐week period between pollination and seed release. The species' seeds are reputed to vary in longevity in dry storage, with *Dact. fuchsii* suggested to be longer‐lived than *Disa uniflora*. Many attempts have been made to store or germinate *Disa uniflora in vitro*, with only a few being successful (Thornhill & Koopowitz 1992; Thompson *et al*. 2006, 2007). Using these model species, this work examined lipid reserve deposition in *Disa uniflora* and *Dact. fuchsii*, from pollination to seed release, germination ability and the relationship between types of FAs and the water content in at least five stages of seed maturation, as well as how seeds behave when stored at different temperatures with distinct moisture content (MC). The hypotheses tested are that interspecies variation in seed FA during development impacts germination efficiency and mature seed storability.

## MATERIALS AND METHODS

The *Dact. fuchsii* are small plants native to England with beautiful inflorescences. Two populations close to the Millennium Seed Bank (51.068870, −0.087512; 51.067524, −0.088718) were selected for the study. Healthy and vigorous plants were chosen, and 10–15 flowers, at most, were pollinated. The upper and lower flowers were pruned to prevent excessive capsule production per plant and to ensure uniform pollination times. Each plant was tagged with an aluminium foil or coloured plastic tag with the pollination date.

*Disa uniflora* is a small plant native to South Africa from Cape Province, with relatively big bright red‐orange flowers. The Royal Botanic Gardens, Kew, purchased a small population of 40 plants and sent them to the Millennium Seed Bank (Wakehurst Place), where they were maintained in the greenhouse (51.067914, −0.090343). They grew under shade, in a wet compost to reflect the native habitat. Two flowers per plant, at most, were pollinated and tagged with aluminium foil bearing the pollination date to ensure the correct harvest time. The genetic relation among plants was examined; neither *Dact. fuchsii* nor *Disa uniflora*, but plants were pollinated with the pollen of the other plant to avoid endogamy.

Capsules were collected with a scalpel near the main flower stalk and placed in a plastic dish (60 × 15 mm), with at least 10 replicates for *Dact. fuchsii* and five for *Disa uniflora*. These capsules were split into two portions: one was sent for germination and tetrazolium (Tz) testing (Hosomi *et al*. 2012; Custodio *et al*. 2016), and the second was used for Differential Scanning Calorimetry (DSC) analysis; moisture content, lipid determination by Gas chromatography–Mass Spectrometry (GC–MS). Capsules were harvested at 14, 28, 38, 42, 48 and 55 days, and at 10, 20, 27, 33 and 42 days after pollination (DAP), respectively, for *Dact. fuchsii* and *Disa uniflora* to accommodate both experiments and the different seed phases from pollination until the start of the maturation.

### Storage after harvesting

Seeds were divided into 24 samples. Half of the samples were conditioned at 4.5% RH [regenerated silica gel (Hay *et al*. 2008)] and the other half at 82.3% RH (Hay *et al*. 2008), to have different conditions for storage in germplasm bank; one with very dried seeds without any notable metabolism, and other with seeds with moisture that allows respiration and deterioration due to oxidative metabolism.

The tubes with screw caps and silicone seals at the lid. After 1 week, the tubes were tightly closed, placed in aluminium‐plastic laminated foil bags and stored in climatic chambers at 5 and 30 °C. Germination, tetrazolium evaluation and fatty acids determination were made monthly during 3 months, split into two portions: one was sent to germination and tetrazolium (Tz) testing (Hosomi *et al*. 2012; Custodio *et al*. 2016), and the second was used for Differential Scanning Calorimetry (DSC) analysis, moisture content and lipid determination by Gas Chromatography–Mass Spectrometry (GCMS).

### Differential scanning calorimetry (DSC) analysis

Seeds were placed in weighted pans, crimp‐sealed and weighed on a UMX2 Ultra‐Microbalance (±0.1 μg, Mettler Toledo, Singapore) to determine the precise sample quantity, then submitted for DSC analysis in a DSC‐7 (Differential Scanning Calorimetry, Perkin Elmer, Shelton, USA). The sequence of cooling and warming conditions was: 1 min at 25 °C, at a cooling rate of −10 °C per min until −100 °C, held for 1 min at −100 °C, followed by warming from −100 °C, at a heating rate of 10 °C per min until 50 °C, where the temperature was held for 1 min at 50 °C. Three replicates were made for each treatment.

DSC runs were run at each harvest point and 1 week after the seeds were equilibrated for storage for both species. Two repetitions were made per treatment.

### Moisture content determination

Following the DSC runs, the pans were perforated and placed in a drying oven at 103 °C for 17 h. The pans were placed over silica gel, left to cool and reweighed in a UMX2 Ultra‐Microbalance (±0.1 μg, Mettler Toledo, Singapore). Moisture content was calculated by the difference in the seed mass. Two repetitions were made per treatment.

### Isotherm confection for *Dactylorhiza fuchsii* and *Disa uniflora* seeds

*Disa uniflora, Dact. fuchsii* and lettuce (*Lactuca sativa* L.) cv Grand Rapids, as an internal control, were conditioned in triplicate in aluminium trays previously weighted and equilibrated for 3 days at 20 ± 1 °C in each one of the different relative humidity obtained with silica gel (4.5%RH) and lithium chloride [13.1, 31.3, 47.4, 58.8, 74.1, 82.3, 94, 95.6 e 97.1%RH, (Hay *et al*. 2008, 2010)] and pure water (~100%). After each condition, the trays were reweighed and transferred to the next. Ultimately, the trays were reweighed and placed in a drying oven at 103 °C for 17 h. The pans were placed over silica gel, left to cool and weighed on a UMX2 Ultra‐Microbalance (±0.1 μg, Mettler Toledo, Singapore). Moisture content was calculated from the difference in seed mass. Two repetitions were made per treatment.

### Fatty acid transesterification

Three seed samples per maturation phase or storage period (10 mg each) were placed in 20 ml glass vials with 1 ml toluene containing 50 mg L^−1^ butylated hydroxytoluene (BHT) as an antioxidant. As an internal standard, 10 ml of 10 mg mL^−1^ heptadecanoic acid was added. The samples were incubated for 4 h at room temperature. Fatty acids were methylated with 2 mL of 1% (v/v) sulphuric acid in methanol (Cavonius *et al*. 2014) overnight at 50 °C with constant shaking at 150 rpm. The fatty acid methyl esters (FAMEs) were isolated by partitioning with 5 mL hexane and 5 mL NaCl (5%, w/v). The organic phase was transferred to a clean vial and the aqueous phase was washed with another 5 ml of hexane. The organic phase was removed carefully to avoid aspirating seeds into the Pasteur pipette. The organic phases were combined and dried at 458C under a stream of nitrogen gas. The residue was dissolved in 1 mL of hexane and transferred to 2‐mL autosampler vials for GCMS analysis.

### Gas chromatography–Mass spectrometry (GCMS)

Three samples of FAMEs were separated by gas chromatography (GC, Thermo Finnigan Trace GC Ultra, Milan, Italy) using a FAMEWAX column (30 m length, 0.25 mm internal diameter, 0.25 mm film thickness (df); Thames Restek UK Ltd, Saunderton, Buckinghamshire, UK) running a temperature program (initial temperature 70 °C; 20 °C min^−1^ until 195 °C; 5 °C min^−1^ until 240 °C; 10 min at 240 °C) with helium as the carrier gas (constant flow rate of 1 ml min^−1^). The compounds were detected by mass spectroscopy (MS, Thermo Finnigan Trace DSQ; ionisation energy 70 eV, scanning frequency m/z 10–500 at 0.3 s) and identified by comparison with the NIST mass spectral database (National Institute of Standards and Technology, Gaithersburg, Maryland, USA) and analytical standards (FAME Mix C4‐ C24, Supelco, Bellefonte, Pennsylvania, USA). FAME quantification was performed using quantitative standard curves (FAME Mix GLC‐10, −30 and −50, Supelco). Three replicates of 10 mg dry weight were used. The data obtained were used to calculate the Peroxidation Index (PI) and the Double Bond Index (DBI) (Pamplona *et al*. 1998). The data were expressed in percentage to make the understanding easier.

### Germination

The capsules were disinfected in a Sodium Dichloroisocyanurate solution (5 g l^−1^) for 10 min and washed three times in autoclaved distilled water. They were cut with forceps and a scalpel, and some seeds were dispersed in the medium. They were disinfected when they were mature (Machado‐Neto & Custódio 2005). Seeds were germinated in three different modifications of MS media (Murashige & Skoog 1962); major and minor salts, vitamins and organics were used at half strength, and sugar source varied from without sucrose (MS‐s), with sucrose (MSS, 20 g L^−1^) or with fructose (MSF, 20 g L^−1^). All media were adjusted to a pH of 5.7 and then autoclaved at 121 °C and 1 atm. MSF was prepared at half volume and autoclaved without sugar. Fructose was filtered, sterilised and added to the medium when it became warm, and the volume was completed with warm autoclaved distilled water. Media were dispensed in Falcon dishes (60 × 15 mm) and kept in the refrigerator until use. Three petri dishes, with at least 150 seeds, were used per replicate per treatment. Seeds were considered germinated when the embryo broke the testa and turned green, phase 1 according to Seaton & Ramsay (2005).

Falcon dishes sown with the seeds were kept in climatic chambers. *Disa uniflora* seeds were germinated at 25 °C with 16 h of light and *Dact. fuchsii* at 25/15 °C with 8 h of light at high temperature.

### Viability (Tetrazolium test)

Seeds were assayed since the beginning, until 48 and 33 days for *Dact. fuchsii* and *Disa uniflora* (Hosomi *et al*. 2011), and the last ones (55 and 42, respectively) and after equilibrium was done in a different way (Custodio *et al*. 2016) due to the presence of a carapace in *Dact. fuchsii* and a thick testa in *Disa uniflora*. Three slides per treatment with at least 150 seeds of each treatment were used.

### Weight and length of capsules

Ten capsules of *Dact. fuchsii* and five of *Disa uniflora* were collected at each harvest time. They were cleaned of flower residues and measured with a digital calliper rule for length and diameter. Capsules were weighed in a UMX2 Ultra‐Microbalance (Mettler Toledo ±0.1 μg).

### Statistical treatment and analysis

Data from capsule dimensions and MC were evaluated as averages ± standard deviations. Germination and viability were compared by an HSD Tukey's test and presented as mean ± standard deviation. Viability and germination of *Disa uniflora* were submitted to a correlation analysis. DSC data were not submitted for statistical analysis. GC–MS data were analysed using ANOVA and *t*‐test (*P* < 0.05) for each species and fatty acid, comparing the harvesting dates.

## RESULTS

There were changes in the morphological parameters of the capsules during development. The length and diameter of capsules increased by 45 and 40%, respectively, for *Dact. fuchsii* (Fig. 1A) and by 39 and 69%, respectively, for *Disa uniflora* (Fig. 1B).

**Fig. 1:**
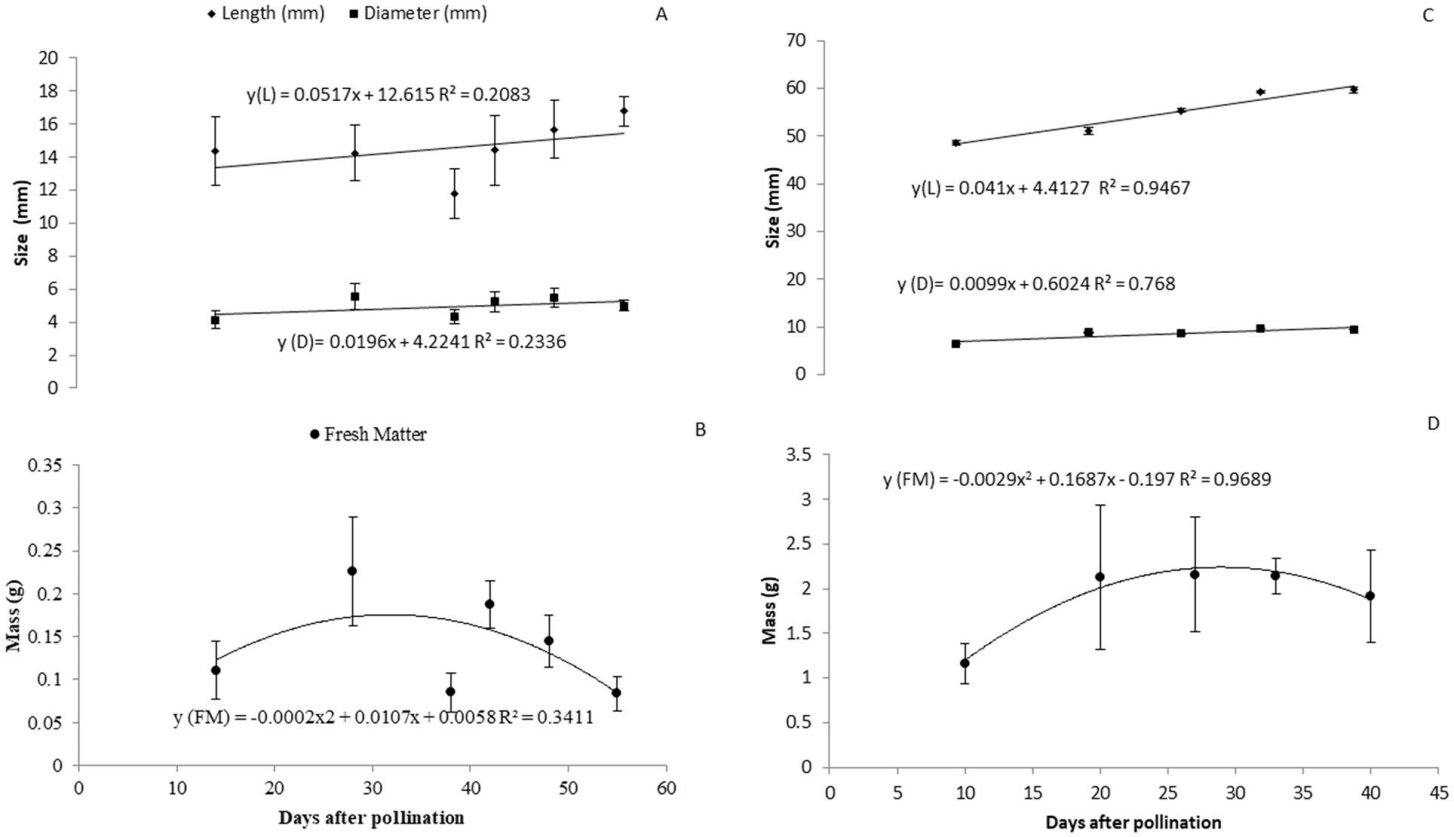
Capsule parameters: diameter (D) and length (L) (A, B), fresh matter (FM) and moisture content (MC) (C, D) of two orchid species – *Dact. fuchsii* (A, C) and *Disa uniflora* (B, D). Vertical bars are ± standard deviation.

Whilst capsule size systematically increased over development (Fig. 1A,C), capsule weight reached a maximum around 28 days after pollination (DAP) in both species (Fig. 1B,D). The decrease in weight later in development is likely explained by the loss of water as the capsules started to dehisce. At capsule maturity, the seeds had almost 70% and 30% of dry matter for *Dact. fuchsii* and *Disa uniflora*, respectively.

Differential scanning calorimetry (DSC) cooling and warming thermograms (Fig. 2A–C for *Dact. fucshii* and 2D and E for *Disa uniflora*) revealed lower seed moisture during maturation, as indicated by the decrease in freezing and melting peaks around 0 to −10 °C, corresponding to water transitions (Fig. 2A,B).

**Fig. 2:**
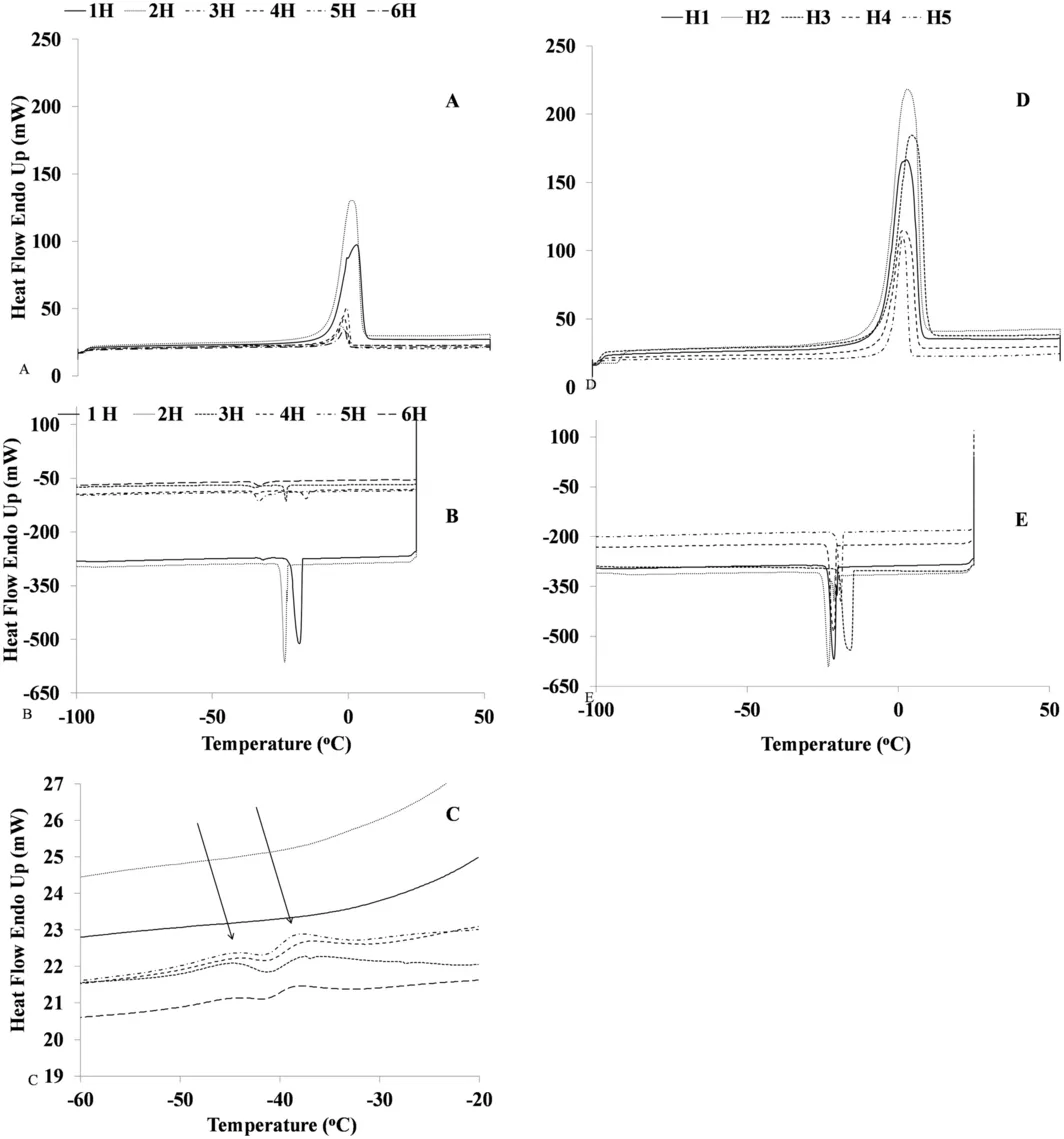
Differential scanning calorimetry of two orchid species – *Dact. fuchsii* seeds warming cycle (A) and cooling cycle (B), and warming cycle detail (C) between −60 and −20 °C. *Disa uniflora* warming cycle (D) and cooling cycle (E). The black arrows in Fig. 2C are putative propyl esters of linolenic acid (C18:2) and oleic acid (C18:1). 1H, 2H, 3H, 4H, 5H and 6H are the dates of harvest for *Dact. fuchsii* (14, 28, 38, 42, 48 and 55 DAP ‐ A, B and C) and H1, H2, H3, H4 and H5 for *Disa uniflora* (10, 20, 27, 33 and 42 DAP – D and E).

Figure 2C shows details of the warming cycle of *Dact. fucshii* seeds, showing an increase in peak signals between −60 and −20 °C for the last four capsule collections. This indicates that in low‐moisture content seeds, enough lipids accumulated later in development to be detected. That in low moisture content seeds, there were enough lipids accumulated later in development e location of the transition events are putatively assigned to propyl esters of linolenic acid (C18:2) and oleic acid (C18:1). On the other hand, *Disa uniflora*, still shows a large amount of moisture content even in the last capsule collection (42 DAP—Figs. 1D and 2D), such that any lipid accumulation in the seed is potentially masked by the water transition signal.

Biochemical analysis showed that seed FA deposition differed between the two species during capsule development (Table 1). For *Dact. fuchsii*, since the first capsule collection (14 DAP), the most synthesised FA was linoleic (18:2), followed by palmitic (16:0). From the second collection (28 DAP), there was a change, and other FAs started to be stored/synthesised. The highest fraction was of polyunsaturated FAs (PUFA). Monounsaturated FA (MUFA) and SFA were in similar proportions.

**Table 1:** FAME composition (proportional out of 100%) of seeds of two orchid species at different times of maturation.

*Dactylorhiza fuchsii*	14 DAP	28 DAP	38 DAP	42 DAP	48 DAP	55 DAP
Methyl palmitate (C16:0)	21.846 ± 0.492a	15.163 ± 0.521b	12.563 ± 1.362c	12.936 ± 0.451 cd	11.742 ± 0.041d	13.636 ± 0.458c
Methyl stearate (C18:0)	0b	1.592 ± 0.222ab	7.143 ± 9.000ab	2.268 ± 0.214a	2.147 ± 0.054ab	1.779 ± 0.092ab
Methyl arachisate (C20:0)	0d	0d	0.149 ± 0.017bc	0.196 ± 0.035b	0.186 ± 0.003b	0.234 ± 0.005a
Methyl behenate (C22:0)	0c	0c	0.113 ± 0.020b	0.113 ± 0.039b	0.128 ± 0.004ab	0.156 ± 0.007a
Methyl lignocerate (C24:0)	0d	0d	0.054 ± 0.048c	0d	0.103 ± 0.001b	0.136 ± 0.002a
Methyl palmitoleate (C16:1)	0d	0d	0.121 ± 0.024a	0.064 ± 0.058bc	0.039 ± 0.055 cd	0.107 ± 0.010ab
Methyl oleate (C18:1)	0d	19.659 ± 0.524a	14.392 ± 1.33bc	15.255 ± 0.776 b	13.444 ± 0.187c	13.849 ± 0.507c
Methyl octadecenoate* (C18:1)	0d	0.658 ± 0.574c	1.561 ± 0.174a	1.270 ± 0.182ab	0.958 ± 0.034bc	1.310 ± 0.065ab
Methyl 11‐eicosenoate (C20:1)	0c	0c	0.121 ± 0.019ab	0.115 ± 0.038ab	0.109 ± 0.009b	0.145 ± 0.013a
Methyl linoleate (C18:2)	76.423 ± 3.462a	62.655 ± 1.131d	63.564 ± 6.17bcd	67.595 ± 1.623 cd	70.989 ± 0.243b	68.442 ± 1.172bc
Methyl linolenate (C18:3)	1.730 ± 2.997a	0.272 ± 0.472a	0.218 ± 0.025a	0.189 ± 0.044a	0.156 ± 0.016a	0.207 ± 0.023a
Saturated	21.846a	16.755a	20.022a	15.512a	14.307a	15.940a
Unsaturated	78.154a	83.245a	79.978a	84.488a	85.693a	84.060a
MUFA	0.000c	20.318a	16.196b	16.704b	14.549b	15.411b
PUFA	78.154a	62.928c	63.782c	67.784bc	71.145b	68.649bc
Unsaturated/saturated	3.577b	4.968ab	3.994ab	5.447ab	5.990a	5.274ab
PI	81.613a	63.980b	64.623b	68.583b	71.82b	69.450b
DBI	158.137	146.445	143.977	152.463	156.993	152.917

*Disa uniflora*	10 DAP	20 DAP**	27 DAP	33 DAP	42 DAP
Methyl palmitate (C16:0)	41.203 ± 3.019a	30.306	29.222 ± 1.068b	22.186 ± 0.978c	20.430 ± 0.668c
Methyl stearate (C18:0)	1.227 ± 1.736a	1.869	1.394 ± 0.295a	3.098 ± 0.081a	3.278 ± 0.308a
Methyl arachisate (C20:0)	0c	0.363	0c	0.495 ± 0.062b	0.744 ± 0.034a
Methyl behenate (C22:0)	0c	0	0c	0.294 ± 0.110b	0.581 ± 0.149a
Methyl lignocerate (C24:0)	0b	0	0b	0.333 ± 0.120a	0.533 ± 0.176a
Methyl palmitoleate (C16:1)	4.130 ± 1.441a	2.099	0.557 ± 0.788b	0.501 ± 0.164b	0.674 ± 0.061b
Methyl oleate (C18:1)	1.058 ± 1.496c	6.659	7.857 ± 0.507b	12.090 ± 0.138a	12.085 ± 0.279a
Methyl octadecenoate* (C18:1)	11.436 ± 0.478a	11.021	8.998 ± 0.325b	5.865 ± 0.082c	5.620 ± 0.209c
Methyl 11‐eicosenoate (C20:1)	0a	0a	0a	0.068 ± 0.096a	0.095 ± 0.134a
Methyl linoleate (C18:2)	39.296 ± 3.509c	43.657	49.717 ± 0.339b	54.256 ± 1.508ab	55.332 ± 0.883a
Methyl linolenate (C18:3)	1.650 ± 2.334a	4.025	2.255 ± 0.142a	0.815 ± 0.119a	0.630 ± 0.201a
Saturated	42.431a	32.538	30.616b	26.406c	25.565c
Unsaturated	57.569c	67.462	69.384b	73.594a	74.435a
MUFA	16.623a	19.780	17.412a	18.523a	18.473a
PUFA	40.946c	47.682	51.971b	55.071a	55.962a
Unsaturated/saturated	1.357c	2.073	2.266b	2.787a	2.912a
PI	43.010b	51.733333ab	59.667a	60.003a	60.84a
DBI	100.167b	119.057ab	133.533a	135.107a	137.810a6

It was not included in the statistical analysis. Samples with the same letters in the lines are not different by the *t*‐test (*P* < 0.05).

DBI, Double Bond Index; MUFA, monounsaturated fatty acid; PI, Peroxidation Index; PUFA, polyunsaturated fatty acid.

* Unresolved double bond.

** 20 DAP of *Disa uniflora* had few extractable seeds and only one sample could be analysed.

In *Disa uniflora*, FA deposition varied markedly over capsule development time (Table 1). A shift from oleic acid (18:1) with an unresolved double bond to oleic acid was noted from the first to the fifth capsule collection (42 DAP). However, UFA deposition was not higher than in *Dact. fuchsii* (Table 1); rather PUFA storage was highest, followed by SFA and the MUFA. More diverse fatty acid types were found just after the third collection.

The impact of capsule harvesting time on germination is shown in Table 2. No seeds germinated when extracted at the first capsule collection (14 DAP for *Dact. fuchsii* and 10 DAP for *Disa uniflora*). The ability to germinate in *Dact. fuchsii* seeds started just after 28 DAP (Fig. 3A). Germination increased after 42 DAP, reaching 40% in MS with sucrose (MSS). At 55 DAP, no germination occurred, likely due to the imposition of dormancy; however, the tetrazolium test showed that the seeds were highly viable (89%, Fig. 3E). In *Disa uniflora*, the beginning of the ability of seeds to germinate (albeit only 30%) began at 20 DAP, when embryos were supplemented with sugars (Table 2). At 42 DAP, seed germination reached a maximum (around 95%) in all media tested: MS without sucrose (MS‐s), MS with fructose (MSF) or MSS. It was found that, after 27 DAP (Table 2), *Disa uniflora* seeds showed a biphasic pattern of germination with two peaks: one in the first 20 days after sowing, followed by a 14‐day pause and the remaining seeds germinated after 34 days after sowing.

**Table 2:** Seed tetrazolium viability (% ± SD) based on embryo staining and seed germination (% ± SD) at different stages of capsule maturation for *Dact. fuchsii* and *Disa uniflora*.

	culture* medium	days after pollination					
		14	28	38	42	48	55
*Dact. fuchsii*		Germination (%)					
	MSF	0c	10 ± 1.5b	7 ± 1.3bc	10 ± 2.3b	34 ± 10.4a	0c
	MS‐s	0c	1 ± 0.8ab	5 ± 0.9ab	9 ± 1.2a	0c	0c
	MS	0c	5 ± 1.1b	7 ± 3.3ab	10 ± 3.4ab	39 ± 5.3	0c
		Viability (%)**					
		0 b	87 ± 2.6ab	86 ± 2.6ab	88 ± 0.3ab	88 ± 0.9ab	90 ± 3.7a

		10	20	27	33	42
*Disa uniflora*		Germination (%)				
	MSF	0d	20 ± 2.6c	43 ± 13.1b	90 ± 3.8a	88 ± 11.2a
	MS‐s	0c	0c	0c	19 ± 3.0b	96 ± 3.7a
	MS	0e	18 ± 6.6 days	37 ± 9.1c	66 ± 8.8b	94 ± 5.4a
		Viability (%)				
		0 days	19 ± 1.2c	17 ± 2.9c	79 ± 3.1b	89 ± 2.2a
Correlation coefficient		MS × MSF			0.960**	
		MS‐s × MS			ns	
		MS‐s × MSF			ns	
		MS × Viability			0.955*	
		MSF × Viability			0.959**	
		MS‐s × Viability			ns	

Similar lowercase letters in the line did not differ at 5% at Tukey's test.

* Medium – MS ½ strength supplemented with sucrose (MS), fructose (MSF) or without added sugar (MS‐s).

** The significance is (*P* < 0.05).

From 28 DAP, seeds of *Dact. fuchsii* had embryos that were visibly coloured by tetrazolium (Fig. 3A). This staining pattern remained until 48 DAP; however, in the last collection (55 DAP), the testa of the seeds became darker, and the embryo colour was not very clear. We solved this problem by including a scarification step in the staining protocol (Custodio *et al*. 2016). Thereafter, counts could be made in the final collection, and showed 89% viability for *Dact. fuchsii* (55 DAP) and 96% for *Disa uniflora* (42 DAP) (Table 2). There were no significant correlations between MS‐S and the other two medias and with viability (Table 2).

**Fig. 3:**
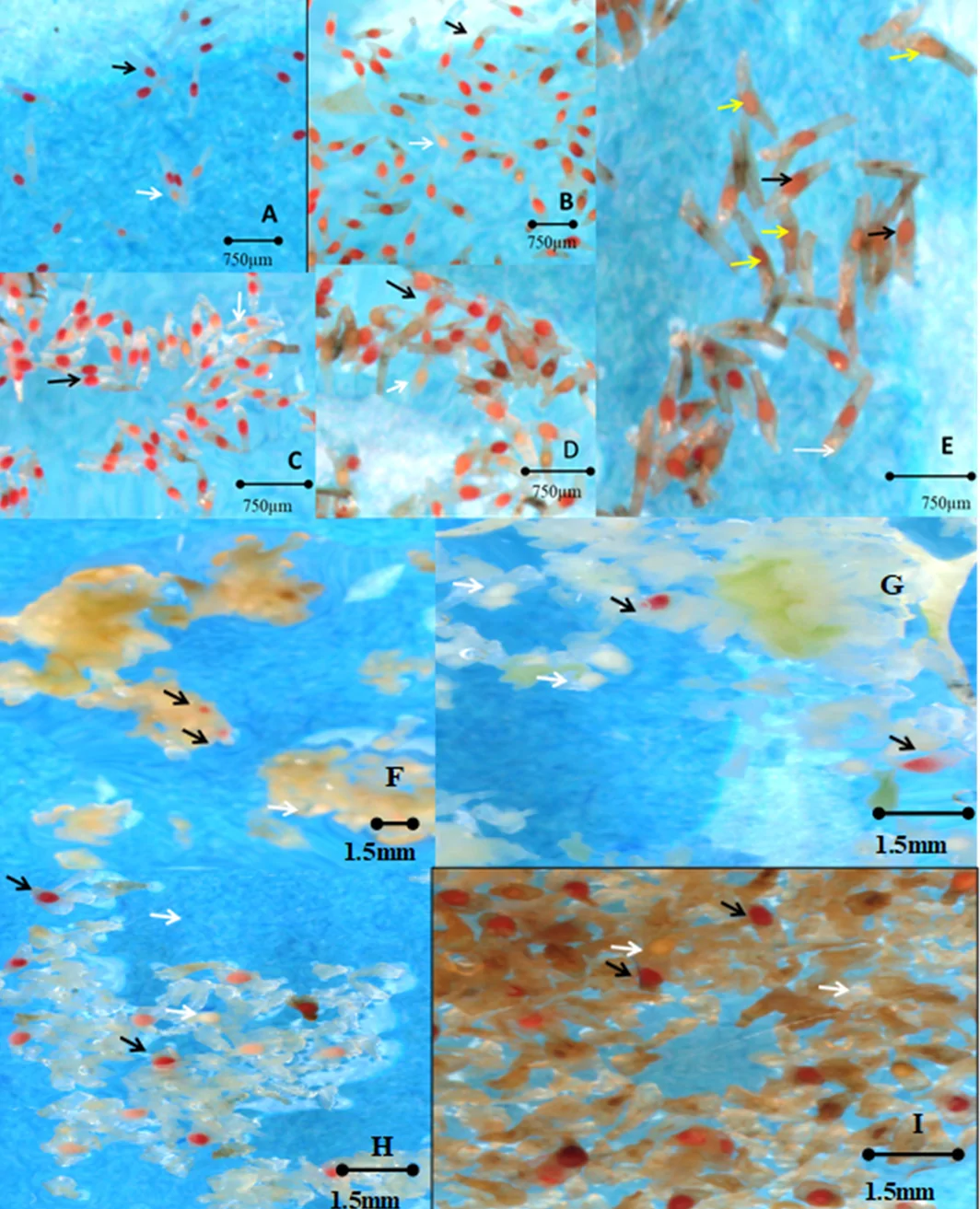
Tetrazolium test of different seed maturation points for *Dact. fuchsii*. (A – 28 DAP, B ‐ 38 DAP; C ‐ 42 DAP, D – 48 DAP and E – 55 DAP) and *Disa uniflora* (F – 20 DAP, G ‐ 27 DAP; H ‐ 33 DAP and I – 42 DAP). Arrows: Black ‐ viable seeds, white ‐ inviable seeds and yellow – possibly low vigour seeds.

Determining the viability of the early collections of *Disa uniflora* (20 DAP) by tetrazolium (Fig. 3F) was hindered by the presence of inert and sticky material in the seed lots. As the capsules matured, there was an increase in the tetrazolium staining intensity (33 and 42 DAP—Fig. 3H,I). Although there were some differences between vital staining and germination at particular DAP (Table 2), there were strong correlations between germination media and the tetrazolium test, with MS and MSF germination highly correlated with Tz colouration. In contrast, the MS–s did not correlate with the Tz test, indicating that sugar, either sucrose or fructose, is essential for germination (Table 2).

Very little debris (hulls, empty or malformed seeds was found in the *Dact. fuchsii* capsule; Fig. 3A). However, *Disa uniflora* capsules contained considerable debris, which was difficult to separate from the seeds and may have contributed to higher estimates of seed moisture content (Fig. 3B). Between species, the seed size and shape were also distinct; *Dact. fuchsii* seeds were found to be filiform, approximately 0.75 mm length *(*Custodio *et al*. 2016 *)* and with an average weight of 1.54 ± 0.28 μg seed^−1^ and *Disa uniflora* were observed to be pear‐shaped, c. 1.16 ± 0.15 × 0.52 ± 0.14 mm, with an average seed weight of 7.2 ± 1.5 μg.

As a prelude to seed storage studies, water sorption isotherms were constructed for the seeds of both species (Fig. 4). The pattern of increasing moisture content with escalating RH was found to be reverse sigmoid. In this pattern, a small increase in RH at low RHs results in a sharp increase in MC. At mid‐range RHs (20–80%), a large increase in RH resulted in a small elevation of MC. Then at high RHs (>80%), seed MC increased steeply. Thus, at 4.5%RH, *Disa uniflora* and *Dact. fuchsii* seeds equilibrated to 4.5% and 4.1% MC, respectively. At 82.3% RH, they equilibrated at 14.5% and 12.2% MC, respectively. Lettuce, which is a known oily seed (c. 37% oil), equilibrated to 2.2 and 11.3% MC under the same two RH conditions.

**Fig. 4:**
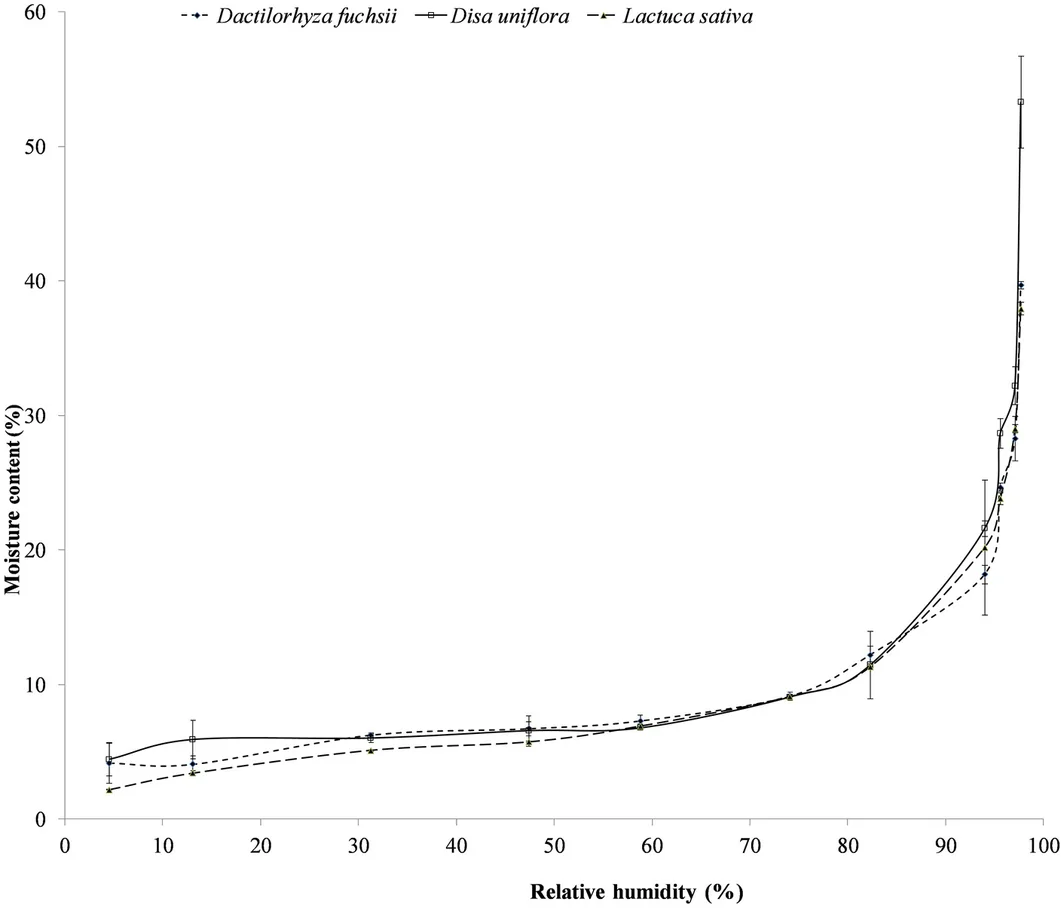
Water sorption isotherms for *Disa uniflora, Dactylorhiza fuchsii* seed and *Lactuca sativa* (lettuce, cv. Grand Rapids) seeds, as a comparison, showing the dependence of seed MC on RH, as controlled by silica gel, lithium chloride solutions and pure distilled water at 20 °C.

Fresh seeds of *Disa uniflora* at the final harvest had an initial MC of 71.4% and germination of 94%. These seeds were equilibrated to 4.5% and 82.3% RH and stored at 5 or 30 °C for up to 90 days (Fig. 2, Table 3). A decrease in seed germination was observed after 60 days of storage under those conditions, with seeds stored at 30 °C after equilibration to 82.3% RH did not germinate after 15 days, indicating rapid ageing.

**Table 3:** Tetrazolium viability (% ± SD) and seed germination (%) of two orchid species: *Dact. fuchsii* and *Disa uniflora* in different storage times after equilibration at two different relative humidities and temperatures.

	eRH (%)	temperature (°C)	time of storage (days)				
			15	30	45	60	90
			viability* (%)				
*Dact. fuchsii*	4.5	5	72 ± 1.8b	85 ± 0.5a	84 ± 0.6a	83 ± 5.8a	85 ± 4.0a
		30	84 ± 0.3a	81 ± 2.6ab	77 ± 0.7b	79 ± 4.0ab	84 ± 1.5a
	82.3	5	79 ± 0.5ab	81 ± 0.8a	70 ± 5.0c	75 ± 1.0bc	76 ± 3.4ab
		30	0	0	0	0	0
			Viability (%)				
*Disa uniflora*	4.5	5	76 ± 3.6a	67 ± 1.4b	80 ± 3.6a	68 ± 4.1b	84 ± 4.5a
		30	60 ± 2.1c	68 ± 2.1bc	78 ± 6.1a	75 ± 4.6ab	78 ± 0.3a
	82.3	5	56 ± 2.7b	62 ± 2.9b	56 ± 5.1b	60 ± 9.6b	70 ± 1.6a
		30	0	0	0	0	0
			Germination (%)**				
	4.5	5	96 ± 0.4a	97 ± 3.1a	88 ± 3.0ab	74 ± 8.1bc	57 ± 12.3c
		30	92 ± 8.6a	86 ± 12.6a	92 ± 3.8a	68 ± 10.7b	57 ± 10.2b
	82.3	5	98 ± 3.6a	92 ± 8.1a	87 ± 11.1a	91 ± 9.4a	59 ± 11.7b
		30	0	0	0	0	0

Similar lowercase letters in the line did not differ at 5% at Tukey's test.

* Medium – MS ½ strength supplemented with sucrose (MS), fructose (MSF) or without added sugar (MS‐s).

** The significance is (*P* < 0.05).

Fresh seeds of *Dact. fuchsii* did not germinate when harvested at 55 DAP, although viability was 90% (Table 2). Viability remained at c. 80% during 90 days' storage at 5 °C and low RH.

High RH and high temperature resulted in the rapid deterioration of the seeds of both species, must probably by cell damage and not by changes in the lipid contents (Tables S1 and S2). At high RH and low temperature, seeds from both species maintained the viability and the germination ability. Nevertheless, the viability of the seeds of both species varied with conditions and over time. *Disa uniflora* exhibited a better storage condition when it was stored at a low MC independent of the temperature. Seeds with a high MC (around 14%) showed decreased viability. The combination of a higher MC and higher temperature was deleterious for both germination and viability for both species (Table 3). Viability retention was higher in *Dact. fuchsii*.

Warming thermograms from the DSC study are shown in Fig. 5. Mature seeds of both species, equilibrated to four RHs, showed somewhat different thermograms. *Disa uniflora* had particularly moist seeds and showed a strong melting peak near zero degrees, assigned to water. No such peak was observable in the drier seeds. The lack of any peaks in the dry material suggests that debris (empty testae) dominated the signal. In contrast, during warming fresh *Dact. fuchsii* seed showed a water peak close to 0 °C, but the drier seed (pretreated with 82% RH and 5% RH) had a melt between c −45 and −15 °C, with a small peak around −40 °C (representing a mixture of the main FA: 18:2, 16:0 and 18:1; Table 1). It is noteworthy that unlike *Disa uniflora*, *Dact. fuchsii* seed lots contained only a few empty testae.

**Fig. 5:**
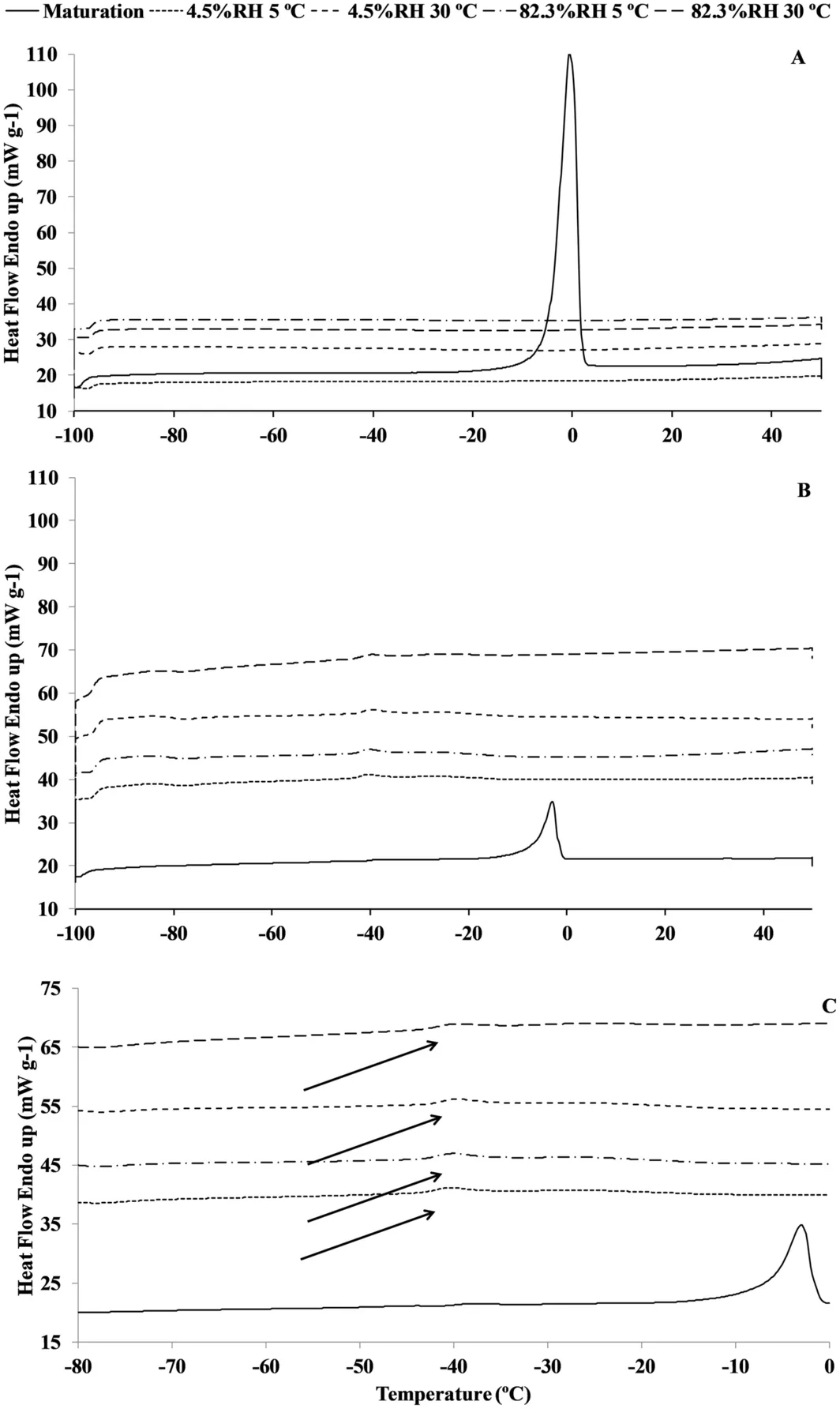
Differential scanning calorimetry of *Disa uniflora* (A) and *Dact. fuchsii* seeds (B) at final maturation and after the initial storage in four distinct conditions. (C) enlargement of a section of *Dact. fuchsii* showing a lipid peak around −40 °C.

The fatty acid composition of the seeds of each species remained relatively stable during storage. Some lipids, such as palmitoleic, lignoceric, arachidic, behenic, linolenic and 11‐eicosenoic, did not appear consistently in the lipid profile but rather at very low concentrations, scattered across the periods; therefore, they are not shown. The principal component was the linoleic acid for both seeds (Tables 1 and 2, Supplemental). For *Disa uniflora*, the second major component was the palmitic acid, followed by the oleic acid (Table 1, Supplemental) for *Dact. fuchsii* (Table 2, Supplemental), either palmitic or oleic, are in the same proportion.

For the principal fatty acids, a difference between the concentration in the final maturation phase and at equilibrium is perceived as fluctuations during storage under different conditions. The main changes occurred at 45 days, when concentrations of palmitic acid increased and those of linoleic acid decreased, but returned to almost the same values by the final period (90 days).

## DISCUSSION

Capsule maturation followed distinct patterns: *Dact. fuchsii* was similar to a field species where seeds must be dried for the approaching cold season, so seeds have a decrease in the water content to around 30% (Fig. 1C) and consequently an increase in the dry matter content, which in the orchid case facilitates wind dispersal of the seeds. Their maturation is similar to that of tomato seeds (Dias *et al*. 2006) or red peppers (Vidigal *et al*. 2011; Ricci *et al*. 2013), both of which are fleshy fruits. Regarding the reserves, the accumulation of unsaturated fatty acids will be an advantage for those species, as these lipids freeze at very low temperatures and thaw at relatively low ones, allowing the maintenance of membrane functionality during the cold season. The presence of peaks in the DSC runs in *Dact. fuchsii* could be the propyl esters of the fatty acids linolenic acid (C18:2) and oleic acid (C18:1) (Fig. 2C) because, according to Knothe & Dunn (2009); Dunn (2012), the pure fatty acids or their esters melt at temperatures around those points. For *Disa uniflora*, even using an amount of seeds similar to that used for *Dact. fuchsii*, and recommended by Colville *et al*. (2016), the water content was so high that it interfered with the analysis (Fig. 2D,E).

For *Disa uniflora* seeds at the capsule dehiscence still showed a high content of water, nearly 70% (Fig. 1D), as a typical species of tropical rainforest or recalcitrant (desiccation‐sensitive) seeds (Barbedo *et al*. 2013; Molizane *et al*. 2020). *Disa uniflora* thrives in moist areas throughout the year and the seeds may not necessarily naturally dry out prior to dispersal. Moreover, the wetter seeds are less prone to longer distance wind dispersal; rather the ‘wet’ seeds may fall and be swept away by water flows. Like recalcitrant (desiccation‐sensitive) seeds (Barbedo *et al*. 2013), seeds of *Disa uniflora* are shed as high moisture content but have a very high level of dehydration down to c. 5% RH (Table 3). Similarly, *Dact. fuchsii* seeds are desiccation tolerant (Table 3). Whilst, some North American orchid species (epiphyte and terrestrial), release their seed at 42% to 46% moisture (Yoder *et al*. 2000) and *Phalaenopsis* at ~50% (Schwallier *et al*. 2011), the overall, evidence suggests orchid species produce drying‐tolerant seeds (Thornhill & Koopowitz 1992; Hay *et al*. 2010; Seaton *et al*. 2018; Fileti *et al*. 2021; Hengling *et al*. 2021; Diantina *et al*. 2022; Francisqueti *et al*. 2024).

The sorption isotherms indicate that seeds of *Dact. fuchsii* and *Disa uniflora* after equilibration to c. 5% RH tolerate drying to 4.1% and 4.5% MC, respectively (Fig. 5, Table 3). Similarly, at high RH (82%), *Disa uniflora* seeds equilibrated to a higher MC than *Dact*. seeds. *fuchsii* seed. The observed differences in MC suggest that *Disa uniflora* seeds likely had lower FA content and higher cellulosic material content (many empty testae). Seeds of *Disa uniflora* are bigger and have a thicker testae than *Dact. fuchsii* and at maturation the seed is sticky, indicative of having hydrophilic material in the seed sample. This was corroborated by the DSC data, where the water peak transition for *Disa uniflora* seed was higher than for *Dact. fuchsii* at the maturation point.

Germination also followed distinct patterns. For *Dact. fuchsii*, germination was low (<40% up to 48 DAP) or non‐existent (0% at 55 DAP) irrespective of the MS media (Murashige & Skoog 1962), even when supplemented with various sugars (sucrose and fructose) or without sugar as a carbon source. However, the tetrazolium test showed that the immature and mature seeds were highly viable (>85%). The difference between germination and viability may be due to the deposition of a coating around the embryo, known as the carapace (Yeung *et al*. 1996; Rasmussen 2011), which acts as protection and hinders germination. Only when the tetrazolium test (Custodio *et al*. 2016) included a scarification path to improve infiltration of the tetrazolium solution did the seed stain fully. *Orchis coriophora*, which also has a carapace, similarly germinates poorly when sown asymbiotically (Bektas *et al*. 2013).

Although mature seeds have the advantage of greater longevity, in some species, they become dormant at maturity (Kitsaki *et al*. 2004; Baskin & Baskin 2021). The lack of germination in *Dact. fuchsii* seed at maturity (Table 2) suggests the imposition of dormancy. This should be an advantage for *Dact. fuchsii* because seeds mature at the end of the summer, when temperatures start to fall and they might benefit from cold treatment over winter prior to germinating in spring. A needed period of cold stratification was verified in *Dact. maculata* (L.) Soó (Andronova *et al*. 2007) and for *Habenaria radiata* (Thunb. Spreng.) [synonym of *Pecteilis radiata* (Thunb.) Raf.] (Takahashi *et al*. 2000); however, thiadiazuron, a plant growth regulator, could overcome the cold requirement for germination (Kim *et al*. 2019). For *Disa uniflora*, on the other hand, the seeds do not exhibit dormancy at the end of the maturation phase. In nature, the plant grows along the edges of water streams in tropical conditions, and its seeds may germinate quickly and form seedlings. A similar situation occurs with *Phalaenopsis* (Schwallier *et al*. 2011), where immature embryos germinated well (above 60%) as early as 90 DAP (~44% of the capsule cycle).

Differences in the ease of asymbiotic germination between the two species were also evident through media preference. *Dact. fuchsii* seed germinates distinctly worse on MS‐s than the other two media (Table 2). In contrast, *Disa uniflora* germinated from the second harvest point (20 DAP) onwards and, contrary to prediction, these seeds germinated and grew in all three media types tested, even in the absence of added sugars (Table 2). Several *Disa* species germinate on a wide range of in vitro media (Thompson *et al*. 2006, 2007). However, we observed that *Disa uniflora* exhibits a distinct germination pattern compared to *Cattleya* seeds (Fileti *et al*. 2021; Hengling *et al*. 2021; Francisqueti *et al*. 2024). Whilst *Cattleya* seeds germinate, quickly break through the testa, and start to synthesise chlorophyll, the *Disa uniflora* seeds imbibe and change from pyriform to spherical. Thereafter, the enlarging embryo becomes more oblong and breaks the integument. Thus, the appearance of seeds in a germination test can vary considerably: (i) globular embryos enveloped by the integument, starting to germinate; (ii) oblong embryos with integument rupture; (iii) oblong embryos with defined leaf primordia; (iv) embryo with leaf primordia and root development. Proper germination is assessed as stage (ii) as in (Yamazaki & Miyoshi 2006), and the later stages (iii and iv) are considered as the embryo‐to‐seedling transitional development. This germination morphology is similar to that proposed for *Dendrobium hookerianum* Lindl. (Paul *et al*. 2012).

For embryo development into seedlings in orchids, an external carbon source is needed for energy. Thus, many authors reported orchid seed germination in a medium containing sucrose (Lee *et al*. 2005; Mahendran *et al*. 2013). No germination data exist for asymbiotic germination without carbon sources. For symbiotic germination, that is, orchid seeds germinating in media without external carbon sources but inoculated with mycorrhizal fungi, several studies have been reported (Batty *et al*. 2001; Bertolini *et al*. 2011; Rasmussen 2011). However, *Disa uniflora* seeds could germinate without added sugar, which would oppose the theory that orchid seeds either need an external sugar source or a mycorrhizal infection (Manning & van Staden 1987; Lee *et al*. 2005, 2006, 2008; Yam & Arditti 2009). Perhaps some degradation of agar occurs during autoclaving (Bhojwani & Razdan 1986), releasing small amounts of soluble sugars into the MS‐s medium.

The main internal source of energy for orchid seed germination may be the accumulated FA, with the composition mix (unsaturated FA *versus* saturated FA) depending on the ecological distribution of the species. Because unsaturated fatty acids crystallise and melt at relatively low temperatures, they are favoured in temperate plants that are regularly exposed to and reestablish after low temperatures. The FA compositional mix in seeds may be important for the storability of the dry seeds. In *Cuphea carthagenensis*, a tropical plant, a high level of saturated medium‐chain fatty acids drives intermediate storage behaviour; that is, seeds die faster when stored cold and at low water content because of lipid crystallisation (Crane *et al*. 2006). *Cuphea carthagenensis* seed lipids melt above 0 °C, which may reflect the saturated FA profile of many tropical species. In contrast, storage of *Dact. fuchsii* and *Disa uniflora* seed in cool, dry conditions is not compromised over 90 days and *Dact. fuchsii* seed has FA that are fluid to below −20 °C. As with many *Cattleya* species that have FA that do not crystallise until the temperature is far below 0 °C (Francisqueti *et al*. 2024), *Dact. fuchsii* seed could avoid crystallisation events during conventional seed bank storage.

Another feature of the FA compositional mix data might indicate that seeds of both the studied species may be bankable. Both species presented a high concentration of MUFA and PUFA (18:1 or higher), leading to the suggestion that these seeds might be classified as orthodox for conventional banking (Liu *et al*. 2006; Mello *et al*. 2010). The suggestion is that unsaturated lipids should not account for <70% of the total lipid amount. Whilst *Disa uniflora* seeds had a higher percentage of saturated FA than *Dact. fuchsii*, both contain >70% unsaturated FAs, being particularly rich at maturation in linoleic acid (18:2). However, *Disa uniflora* had a slightly higher amount of palmitic acid (16:0) and it would be interesting if future studies could explore the thermal properties of these seeds at sub‐zero temperature. It seems relevant also to investigate orchid seed lipid stability over longer term dry, cold storage, as previously examined with *Cattleya* (Pritchard & Seaton 1993; Francisqueti *et al*. 2024) and *Arundina graminifolia* (Dai *et al*. 2026) seeds, as a way to indicate conservation potential. As noted here, immature seeds are not a good source of material for conventional seed banking.

FA concentrations differed between species. Convergence was observed in linoleic acid (18:2), a UFA, which has the highest concentration in both species, but in different amounts (~68% in *Dact. fuchsii* and ~55% in *D. uniflora*). The other most abundant UFA was the oleic (18:1) (~14% and ~12, respectively). The most abundant SFA was palmitic acid (16:0), with ~14% and ~20% in each species. These concentrations may help in seed storage. The evolutionary history of orchids begins in the northern hemisphere, in a hot climate (Ramírez *et al*. 2007), which should impact the kind of storage lipids. Palmitic acid is frequently found in TAGs (Ohlrogge & Browse 1995) and plays an important role in them. It should be expected that plants that did not experience freezing in their evolutionary story might have a high amount of these kinds of acids; however, as the climate alterations throughout the eras, there is a change in the accumulation of FA from SFA to PUFA, which allows seeds to freeze without dying with temperature increasing in the next seasons. Although PUFAs are more prone to ROS‐mediated damage, it seems that such conditions (high temperatures and RH) might be tough enough to harm the seeds. Francisqueti *et al*. (2024) found that high oleic acid content may be detrimental to tropical orchid seed, suggesting that both species here belong to an older lineage (Orchidoidae) (Wang *et al*. 2024) and retain a high content of this acid.

## CONCLUSIONS

In geophytes like *Dact. fuchsii* and *Disa uniflora*, the maturation of fruits and seeds is similar to that of plants growing in the field, savannah‐like environments or tropical forests at the maturation point. The two species behave as orthodox seeds. There were differences in lipid accumulation patterns, which could reflect a larger gradient within such a family, and this may help in taking decisions before storage. Seeds must be very well dried to ensure proper storage, and the tetrazolium test could be used to monitor seeds with difficult germination.

## AUTHOR CONTRIBUTIONS

Conceptualization: NBMN, CCC, PTS, TRM and HWP; methodology: NBMN, CCC and LC; software: NBMN, CCC and LC; validation: NBMN, CCC and LC; formal analysis: NBMN, CCC and LC; investigation: XX; resources: HWP and TRM; data curation: NBMN, CCC and LC; writing – original draft preparation: NBMN, CCC, PTS, TRM, LC and HWP; writing – review and editing: NBMN, CCC; visualisation: XX; supervision: HWP and TRM; project administration: NBMN, CCC and TRM; funding acquisition: HWP and TRM. All authors have read and agreed to the published version of the manuscript.

## FUNDING INFORMATION

This research was funded by CNPq grants 245783/2012‐1 and 245777/2012‐1.

## CONFLICT OF INTEREST STATEMENT

The authors declare no conflicts of interest.

## Supporting information


**Table S1.** *Disa uniflora* major lipids seed composition (%) during storage in four distinct conditions.
**Table S2**. *Dactylorhiza fuchsii* major lipids seed composition during storage in four distinct conditions.

## Data Availability

All data will be available upon request to the corresponding author.
