# Supplementary material for: Lipidomics of orchid seeds: from development to storage
Source: Plant Biol (Stuttg). 2026 Sep 23;28(7):2255–69. doi: 10.1111/plb.70295 (PMC13750338; doi:10.1111/plb.70295)
Supplement: Supplementary file 1 [file PLB-28-2255-s001.docx]

**Table S1** – *Disa uniflora* major lipids seed composition (%) during storage in four distinct conditions.

|  |  | Time of storage (days) | | | | |
| --- | --- | --- | --- | --- | --- | --- |
|  |  | 15 | 30 | 45 | 60 | 90 |
|  |  | Temperature of storage (5°C) | | | | |
| RH (%) | FAME (%) |  |  |  |  |  |
|  | Methyl palmitate (C16:0) | 19.096 ±0.820b | 19.164 ±1.459b | 22.380 ±1.661a | 20.882 ±1.688ab | 20.550 ±0.506ab |
| 4.5 | Methyl stearate (C18:0) | 2.188 ±0.451a | 2.600 ±0.561a | 2.023 ±0.320a | 1.900 ±0.408ab | 0.893 ±0.774b |
|  | Methyl oleate (C18:1) | 9.709 ±0.481 | 10.264 ±0.643 | 10.090 ±0.572 | 9.613 ±1.465 | 10.823 ±0.216 |
|  | Methyl octanodecenoate* (C18:1) | 4.455 ±0.367 | 4.531 ±0.288 | 4.518 ±0.218 | 4.443 ±0.464 | 4.221 ±0.409 |
|  | Methyl linoleate(C18:2) | 64.551 ±2.120a | 61.838 ±1.498ab | 60.608 ±0.275b | 62.465 ±1.914ab | 63.513 ±0.777a |
|  | MUFA^&^ | 14.165 | 15.265 | 14.727 | 14.366 | 15.044 |
|  | PUFA^&^ | 64.551 | 62.162 | 60.692 | 62.585 | 63.513 |
|  | Saturated^&^ | 21.285 | 22.573 | 24.581 | 23.048 | 21.442 |
|  | Unsaturated^&^ | 78.715 | 76.958 | 75.300 | 76.642 | 78.557 |
|  | Unsaturated/Saturated^&^ | 3.698 | 3.409 | 3.063 | 3.325 | 3.664 |
|  | PI | 68.090a | 66.303ab | 64.460b | 66.293ab | 67.277a |
|  | DBI | 143.266a | 139.917ab | 136.197b | 139.657ab | 142.073a |
|  | Methyl palmitate (C16:0) | 18.319 ±0.625b | 20.310 ±1.025ab | 21.578 ±1.481a | 21.665 ±1.910a | 19.099 ±2.672ab |
| 82.3 | Methyl stearate (C18:0) | 2.488 ±0.183a | 2.279 ±0.373ab | 1.907 ±0.302ab | 1.605 ±0.283ab | 1.349 ±0.156b |
|  | Methyl oleate (C18:1) | 10.177 ±0.624ab | 9.815 ±0.645ab | 10.025 ±0.699ab | 9.480 ±0.193b | 10.968 ±0.642a |
|  | Methyl octanodecenoate* (C18:1) | 4.377 ±0.377 | 4.468 ±0.226 | 4.615 ±0.219 | 4.152 ±0.800 | 4.272 ±0.456 |
|  | Methyl linoleate(C18:2) | 64.132 ±1.164a | 62.466 ±0.658ab | 61.251 ±1.693b | 63.098 ±0.634ab | 64.312 ±2.156a |
|  | MUFA^&^ | 14.554 | 14.786 | 14.756 | 13.631 | 15.240 |
|  | PUFA^&^ | 64.292 | 62.466 | 61.650 | 63.098 | 64.312 |
|  | Saturated^&^ | 21.154 | 22.748 | 23.594 | 23.270 | 20.448 |
|  | Unsaturated^&^ | 78.846 | 76.749 | 76.289 | 76.730 | 79.552 |
|  | Unsaturated/Saturated^&^ | 3.727 | 3.374 | 3.233 | 3.297 | 3.890 |
|  | PI | 67.257 | 65.653 | 65.603 | 64.947 | 64.597 |
|  | DBI | 141.960 | 138.433 | 138.417 | 137.270 | 139.203 |

**Table S1**– Cont, *Disa uniflora* major lipids seed composition (%) during storage in four distinct conditions

| Temperature of storage (30°C) | | | | | | |
| --- | --- | --- | --- | --- | --- | --- |
|  | Methyl palmitate (C16:0) | 19.285 ±0.840 | 20.968 ±2.058 | 21.481 ±0.389 | 22.036 ±1.168 | 20.856 ±0.207 |
| 4.5 | Methyl stearate (C18:0) | 2.286 ±0.258 | 2.346 ±0.409 | 2.032 ±0.192 | 1.807 ±0.320 | 1.537 ±0.245 |
|  | Methyl oleate (C18:1) | 10.380 ±0.663 | 10.044 ±0.914 | 9.957 ±0.700 | 10.174 ±0.584 | 11.153 ±0.608 |
|  | Methyl octanodecenoate* (C18:1) | 4.519 ±0.175 | 4.367 ±0.356 | 4.456 ±0.280 | 4.589 ±0.281 | 4.763 ±0.575 |
|  | Methyl linoleate(C18:2) | 63.531 ±1.219 | 61.518 ±1.227 | 62.002 ±1.569 | 61.078 ±0.760 | 61.596 ±1.394 |
|  | MUFA^&^ | 14.898 | 14.707 | 14.413 | 14.890 | 16.010 |
|  | PUFA^&^ | 63.530 | 61.74799 | 62.001 | 61.15309 | 61.596 |
|  | Saturated^&^ | 21.571 | 23.54525 | 23.585 | 23.95725 | 22.39 |
|  | Unsaturated^&^ | 78.429 | 76.15865 | 76.415 | 75.91622 | 77.512 |
|  | Unsaturated/Saturated^&^ | 3.63 | 3.23 | 3.24 | 3.17 | 3.46 |
|  | PI | 68.090 | 66.163 | 65.737 | 66.507 | 68.120 |
|  | DBI | 143.297a | 139.720ab | 138.453b | 139.783 | 143.863a |
|  | Methyl palmitate (C16:0) | 20.099 ±1.204a | 19.717 ±2.477a | 22.193 ±0.475a | 21.496 ±1.026a | 15.769 ±0.907b |
| 82.3 | Methyl stearate (C18:0) | 2.180 ±0.310 | 1.751 ±1.519 | 2.066 ±0.135 | 1.767 ±0.229 | 2.132 ±0.242 |
|  | Methyl oleate (C18:1) | 10.151 ±0.179b | 10.459 ±0.200b | 10.323 ±0.116b | 9.970 ±0.640b | 13.189 ±0.179a |
|  | Methyl octanodecenoate* (C18:1) | 4.287 ±0.253 | 4.589 ±0.179 | 4.575 ±0.088 | 4.443 ±0.481 | 5.074 ±0.087 |
|  | Methyl linoleate(C18:2) | 63.017 ±0.086ab | 62.069 ±0.954ab | 60.531 ±0.389b | 62.323 ±0.280ab | 63.711 ±0.952a |
|  | MUFA^&^ | 14.438 | 15.511 | 15.061 | 14.4131 | 18.3881 |
|  | PUFA^&^ | 63.017 | 62.337 | 60.531 | 62.3231 | 63.711 |
|  | Saturated^&^ | 22.545 | 22.151 | 24.408 | 23.2631 | 17.901 |
|  | Unsaturated^&^ | 77.455 | 77.386 | 75.429 | 76.737 | 81.979 |
|  | Unsaturated/Saturated^&^ | 3.436 | 3.493 | 3.090 | 3.299 | 4.5793 |
|  | Pi | 66.627ab | 66.483ab | 64.297b | 65.923ab | 68.307a |
|  | DBI | 140.473b | 140.453b | 136.123b | 139.060b | 145.810a |

*unresolved double bond. ^&^ not submitted to analysis. Samples with the same or without lowercase letters in the lines are not different by the *t*-test (p<0.05).

**Table S2** – *Dactilorhyza fuchsii* major lipids seed composition during storage in four distinct conditions.

|  |  | Time of storage (days) | | | | |
| --- | --- | --- | --- | --- | --- | --- |
| RH (%) | FAME (%) | **15** | **30** | **45** | **60** | **90** |
|  |  | Temperature of storage (5°C) | | | | |
| 4.5% | Methyl palmitate (C16:0) | 11.335 ±0.904d | 11.878 ±0.083cd | 14.386 ±0.704a | 12.607 ±0.445bc | 13.302 ±0.497ab |
|  | Methyl stearate (C18:0) | 1.874 ±0.484 | 1.806 ±0.028 | 1.952 ±0.343 | 1.882 ±0.123 | 1.537 ±0.277 |
|  | Methyl oleate (C18:1) | 13.490 ±1.075ab | 13.095 ±0.670ab | 14.507 ±0.680a | 13.835 ±0.394ab | 13.365 ±0.575ab |
|  | Methyl octadecenoate* (C18:1) | 1.093 ±0.088 | 1.063 ±0.300 | 1.185 ±0.115 | 1.030 ±0.416 | 1.152 ±0.363 |
|  | Methyl linoleate(C18:2) | 71.928 ±1.388a | 71.927 ±0.917a | 67.773 ±1.752b | 70.802 ±1.693a | 70.449 ±0.975a |
|  | MUFA^&^ | 14.662 | 14.158 | 15.692 | 15.222 | 14.557 |
|  | PUFA^&^ | 71.993 | 72.050 | 67.871 | 70.846 | 70.501 |
|  | Saturated^&^ | 13.345 | 13.792 | 16.437 | 13.932 | 14.942 |
|  | Unsaturated^&^ | 86.655 | 86.208 | 83.563 | 86.068 | 85.058 |
|  | Unsaturated/Saturated^&^ | 6.49 | 6.25 | 5.08 | 6.18 | 5.69 |
|  | Pi | 75.723a | 75.713a | 71.897b | 74.697a | 74.190a |
|  | DBI | 157.713a | 158.380a | 151.530b | 156.957a | 155.610a |
| 82.3% | Methyl palmitate (C16:0) | 13.549 ±0.013b | 11.512 ±0.180c | 15.250 ±0.822a | 12.350 ±0.587ab | 12.384 ±0.513ab |
|  | Methyl stearate (C18:0) | 1.401 ±0.071b | 1.930 ±0.151ab | 2.228 ±0.341a | 1.802 ±0.080ab | 1.657 ±0.239ab |
|  | Methyl oleate (C18:1) | 12.483 ±0.286b | 14.553 ±0.346a | 15.431 ±0.409a | 15.288 ±0.603a | 14.422 ±0.768a |
|  | Methyl octadecenoate* (C18:1) | 0.585 ±0.095 | 0.991 ±0.239 | 0.975 ±0.175 | 1.048 ±0.378 | 0.848 ±±0.279 |
|  | Methyl linoleate(C18:2) | 71.982 ±0.439a | 70.849 ±0.710a | 65.316 ±1.594b | 69.425 ±1.146a | 70.689 ±0.974a |
|  | MUFA^&^ | 13.068 | 15.5701 | 16.655 | 16.360 | 15.270 |
|  | PUFA^&^ | 71.982 | 70.884 | 65.4778 | 69.447 | 70.689 |
|  | Saturated^&^ | 14.950 | 13.546 | 17.867 | 14.193 | 14.041 |
|  | Unsaturated^&^ | 85.050 | 86.454 | 82.025 | 85.807 | 85.959 |
|  | Unsaturated/Saturated^&^ | 5.69 | 6.38 | 4.59 | 6.04 | 6.12 |
|  | Pi | 74.197a | 74.800a | 70.903b | 74.103a | 74.020a |
|  | DBI | 157.873a | 156.950a | 149.670b | 155.980a | 155.713a |

**Table S2** cont – *Dactilorhyza fuchsii* major lipids seed composition during storage in four distinct conditions.

|  |  | Time of storage (days) | | | | |
| --- | --- | --- | --- | --- | --- | --- |
| RH (%) | FAME (%) | **15** | **30** | **45** | **60** | **90** |
|  |  | Temperature of storage (30°C) | | | | |
| 4.5% | Methyl palmitate (C16:0) | 12.211 ±0.505b | 11.804 ±0.130b | 15.369 ±0.834a | 12.362 ±0.343b | 12.698 ±0.387b |
|  | Methyl stearate (C18:0) | 2.078 ±0.318 | 2.021 ±0.328 | 1.851 ±0.239 | 1.717 ±0.340 | 1.607 ±0.309 |
|  | Methyl oleate (C18:1) | 14.114 ±0.604 | 13.705 ±0.640 | 14.447 ±0.777 | 14.468 ±0.275 | 14.311 ±0.272 |
|  | Methyl octadecenoate* (C18:1) | 1.030 ±0.113 | 1.119 ±0.236 | 1.276 ±0.101 | 1.160 ±0.356 | 1.094 ±0.238 |
|  | Methyl linoleate (C18:2) | 70.132 ±1.605a | 70.748 ±1.031a | 66.973 ±0.453b | 70.047 ±1.024a | 69.983 ±0.706a |
|  | MUFA^&^ | 15.226 | 15.001 | 15.723 | 15.683 | 15.498 |
|  | PUFA^&^ | 70.261 | 70.899 | 66.973 | 70.115 | 70.066 |
|  | Saturated^&^ | 14.513 | 14.100 | 17.303 | 14.202 | 14.436 |
|  | Unsaturated^&^ | 85.487 | 85.822 | 82.697 | 85.775 | 85.502 |
|  | Unsaturated/Saturated^&^ | 5.89 | 6.09 | 4.78 | 6.04 | 5.92 |
|  | Pi | 72.500a | 74.810 | 69.807b | 73.560a | 74.503a |
|  | DBI | 155.030a | 157.373a | 147.773b | 155.277a | 155.647a |
| 82.3% | Methyl palmitate (C16:0) | 12.632 ±0.420b | 11.794 ±0.460b | 14.578 ±0.474a | 12.601 ±0.414b | 12.477 ±0.452b |
|  | Methyl stearate (C18:0) | 1.500 ±0.169b | 2.081 ±0.147a | 1.891 ±0.154ab | 1.920 ±0.050ab | 1.583 ±0.292ab |
|  | Methyl oleate (C18:1) | 13.346 ±0.394b | 14.095 ±0.232ab | 15.043 ±0.368a | 15.247 ±0.376a | 14.158 ±0.759ab |
|  | Methyl octadecenoate* (C18:1) | 1.029 ±0.071 | 1.006 ±0.216 | 0.857 ±0.280 | 0.954 ±0.362 | 0.969 ±0.390 |
|  | Methyl linoleate (C18:2) | 71.494 ±0.382a | 70.509 ±0.117a | 67.508 ±1.190 b | 69.167 ±1.205ab | 70.754 ±1.665a |
|  | MUFA^&^ | 14.375 | 15.240 | 15.932 | 16.227 | 15.186 |
|  | PUFA^&^ | 71.493 | 70.611 | 67.508 | 69.167 | 70.754 |
|  | Saturated^&^ | 14.131 | 14.149 | 16.560 | 14.606 | 14.060 |
|  | Unsaturated^&^ | 85.868 | 85.791 | 83.408 | 85.368 | 85.881 |
|  | Unsaturated/Saturated^&^ | 6.08 | 6.06 | 5.04 | 5.84 | 6.11 |
|  | Pi | 75.090a | 74.523a | 71.490b | 73.223ab | 74.547a |
|  | DBI | 157.363a | 156.563a | 150.947b | 154.563a | 156.693a |

*unresolved double bond. ^&^ not submitted to analysis. Samples with the same or without lowercase letters in the lines are not different by the *t*-test (p<0.05).
